# A Case of Successfully Treated Rhinocerebral Mucormycosis: Dental Implications

**DOI:** 10.1155/2010/273127

**Published:** 2011-02-15

**Authors:** Nikolaos Papadogeorgakis, Eleni Parara, Vassilios Petsinis, Christina Vourlakou

**Affiliations:** ^1^Oral and Maxillofacial Surgery Department, Evangelismos General Hospital, 41-44 Ipsilantoust, 106 76 Athens, Greece; ^2^Histopathology Department, Evangelismos General Hospital, 41-44 Ipsilantoust, 106 76 Athens, Greece

## Abstract

This paper presents a case of rhinocerebral mucormycosis in a 22-year-old female patient with type I diabetes mellitus, who was successfully treated with surgery and long-term antifungal medication. The patient had initially been submitted to extraction of an upper third molar by a general dental practitioner but was referred to our department three days postoperatively because of double vision. Immediately following histopathological confirmation of the infection, the patient was administered Amphotericin B and Posaconazole intravenously. Surgical excision of the affected site was relatively conservative. The patient was free of the disease 15 months after initial admission to the hospital and has recently returned for reconstruction. The aim of this paper is to increase the awareness of general dental practitioners regarding uncommon serious conditions in diabetic patients, which may be confused with periodontal or dental diseases.

## 1. Introduction

Mucormycosis is a rare and potentially lethal infection. It is caused by fungi, which may be found in decaying food, in the soil, or other organic matter, such as animal excreta [1]. 

Immunocompromised patients are especially susceptible, and timely diagnosis as well as intervention are of utmost importance for successful management. Because of atypical initial symptoms, such as facial pain, earache, sinus pain, or odontalgia, patients may seek dental treatment initially. Dental care may also precede such an infection, by means of a creation of a postextraction or postcurretage wound, which may be susceptible to fungal infection [2]. The aim of this case report is to present a patient with rhinocerebral mucormycosis who was successfully treated due to timely diagnosis, effective surgical intervention, and intravenous antifungal treatment.

## 2. Case Report

A 22-year-old woman was transferred to the Oral and Maxillofacial Department for facial pain, oedema, and double vision, following extraction of the upper right third molar tooth three days earlier. The patient had initially visited an ear-nose-throat specialist because of diffuse pain of the midface a week previously. They had recommended a dental examination of the upper right molars and premolars. The general dental practitioner had decided to extract the patient's upper right third molar tooth, which was in close contact to the inferior wall of the maxillary sinus, assuming that the symptoms were odontogenic. 

**Figure 1:**
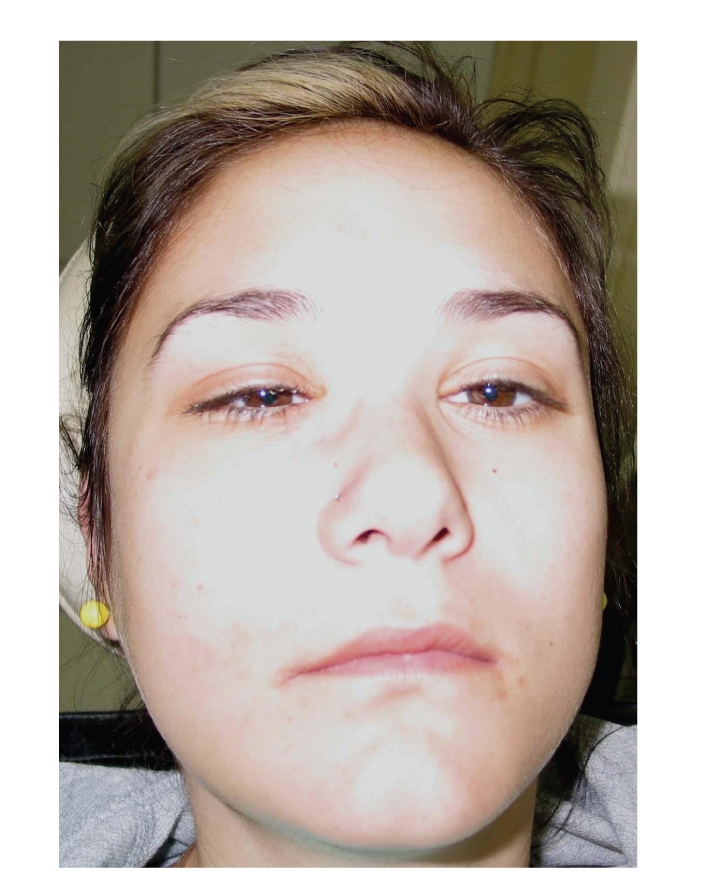


**Figure 2:**
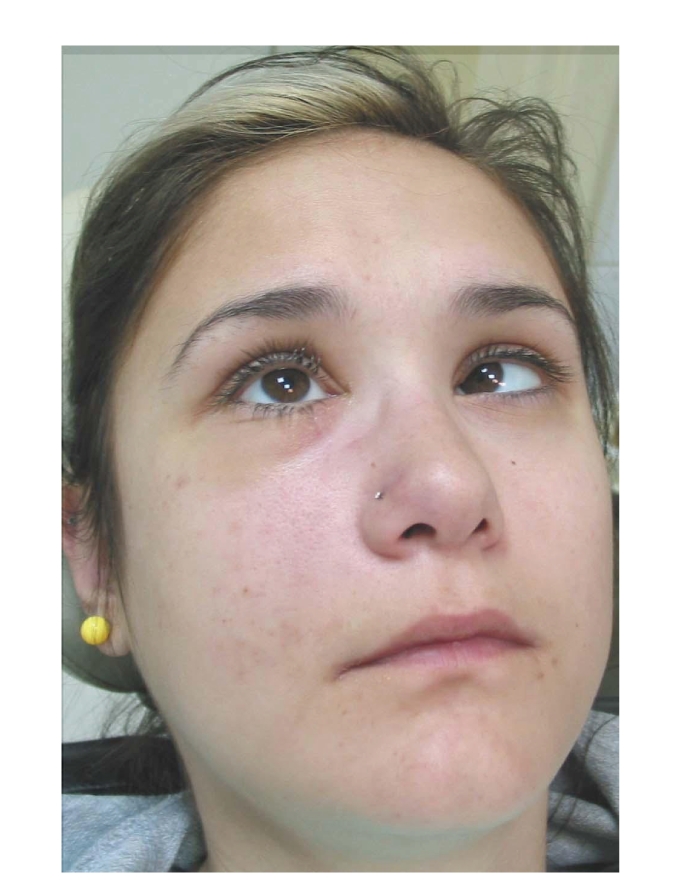


**Figure 3:**
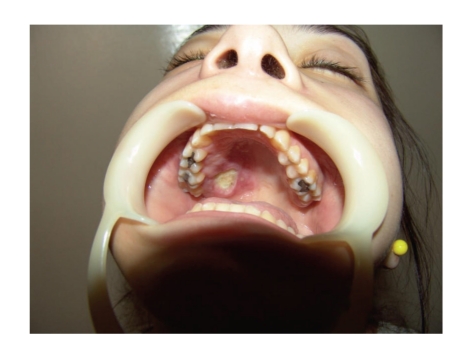


The general dental practitioner had prescribed amoxicillin postoperatively and advised on oral hygiene. However, the pain had gradually deteriorated after the extraction. The patient had been hospitalized for generalized facial oedema and pain in a regional hospital but was transferred to our institution twenty-four hours later, when she developed double vision due to paralysis of the right lateral rectus muscle. The patient's medical history included diabetes mellitus type I, which was poorly controlled. On clinical examination, she presented slight hemifacial oedema of the right side, inward gaze of the right eye because of paralysis of the abduscent nerve (VI cranial nerve), as can be seen in Figures 1 and 2. Neurological examination also revealed hypaesthesia of the area of distribution of the maxillary nerve (2nd branch of the V cranial nerve). The right side of the palate was red with an ulcer of 1 cm diameter, near the second upper molar tooth (Figure 3). 

Generally, the patient was an undernourished young woman, with normal vital signs, and unwilling to provide information regarding her diabetes or the reason for dental care. Undertaken investigations at admittance included blood count, serum electrolytes, urea, kreatinin, glucose, and C-reactive protein. These revealed elevated blood glucose and mild dehydration. Furthermore, glycated haemoglobin (glycohemoglobin, HbA1c) was raised to 12.3%, with the normal range between 4–5.9% (Table 1). Opthalmological examination showed palpebral oedema of the right side, no lesions of the retina or optic nerve, but diplopia at the right outward gaze, which confirmed the paralysis of the right lateral rectus muscle. 

**Figure 4:**
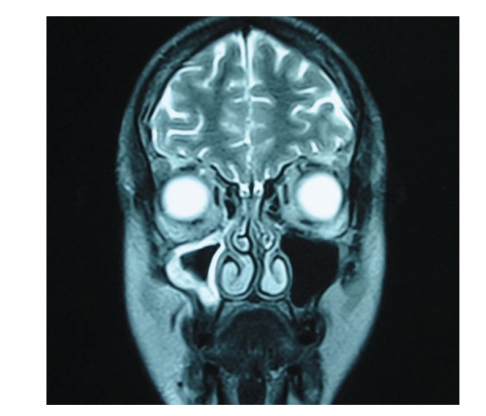


**Figure 5:**
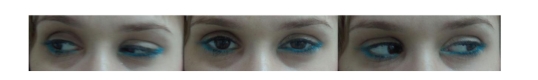


**Figure 6:**
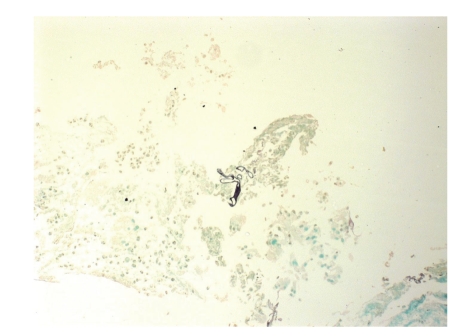


Computerised tomography revealed thickening of the mucosal lining of the paranasal sinuses. Further imaging with magnetic resonance imaging also showed involvement of all paranasal sinuses of the right side, however, without involvement of the central nervous system (Figure 4). A biopsy of the palatal ulcer showed fungal infection by species of Mucorales (Figure 5). The patient was initiated with intravenous antifungal agents (amphotericin B 300 mg qd and posaconazole 200 mg tid), and three days later she underwent subtotal right maxillectomy and reconstruction with an obturator. Thereafter, she continued the antifungal treatment, presenting gradual improvement. The paralysis of the right lateral rectus muscle progressively recovered, so the patient was dismissed from the hospital 2 months later, free of any infection, and has recently returned for definitive reconstruction (Figure 6). During her hospitalization, our patient was also accustomed to more effective blood glucose management (Table 1).

## 3. Discussion

The species of Mucorales invade blood vessels and cause necrosis of vessel walls and mycotic thrombi. While healthy humans are resistant to such an infection, immunocompromised patients are generally much more vulnerable to the angioinvasive hyphal forms of these fungi [1]. Diabetes mellitus, neutropenia, severe trauma, immunosuppression following transplantation of bone marrow, or solid organs are all predisposing factors for mucormycosis [1, 3, 4]. The host-specific condition may however render the host susceptible to different types of the infection.

Mucormycosis is classified according to the anatomic site of occurrence in (1) rhinocerebral, (2) pulmonary, (3) cutaneous, (4) gastrointestinal, and (5) disseminated [5]. 

Rhinocerebral or sino-orbital types are common among diabetic patients, especially those who are poorly controlled. The above-mentioned types of infection may present with various and atypical symptoms of sinusitis [1, 6]. Nasal congestion, headache, earache, or facial pain are some of the most common features, which are not at all characteristic. 

Depending on the affected site, adjacent structures like the orbit or the central nervous system may be involved. Periorbital edema, ophthalmoplegia, or deterioration of vision is of high probability. Similarly, extension to the cavernous sinus may cause cavernous sinus thrombosis [1, 2] or affect cranial nerves. Through the cribriform plate of the ethmoid bone or the supraorbital fissure, the infection may spread intracranially and cause abscesses or sagittal sinus thrombosis [1]. Perineural invasion and spread has also been reported [2, 7]. The affected area is initially clear of any signs, but soon may appear reddish with or without necrotic eschars [1, 4]. Especially intraorally, the palate may present necrotic with large black eschars. Nasal endoscopy is usually necessary to reveal other necrotic lesions [1]. 

Radiographic findings are generally inconclusive and not specific. CT and MRI are within normal limits initially, which are followed by signs of sinusitis, such as congested sinus or thickened mucosal lining. Repetition of the investigations is necessary for close followup of the advancement of the disease. Differential diagnosis mainly includes necrotizing fasciitis [3], especially if facial oedema is present. As the condition rapidly aggravates, timely diagnosis is crucial. A histopathological diagnosis is generally considered more precise than simple culture, as the latter may be unyielding due to the depth of invasion of the infection [1, 8, 9]. 

Successful management depends on timing. As soon as the clinical suspicion has risen, it is imperative to perform a biopsy of the area and initiate intravenous antifungal treatment [1, 2, 9]. Early intervention with ablative surgery is generally recommended [10–14]. 

Especially considering rhinocerebral or rhino-orbital mucormycosis, enucleation of the eye is contemplated as imperative for definitive treatment. Although the Internal Medicine faculty strongly suggested total maxillectomy and removal of the eye, because of the aforementioned evidence, the absence of vision disturbance, severe conjunctival infection, or even optic nerve involvement in a young patient led to a more conservative maxillectomy. Indeed, the patient recovered following long-term antifungal treatment. 

This fact is probably due to early intervention as well as no participation of the contents of the orbit, but cranial nerves. However, signs of involvement of the orbital area should always direct to a total maxillectomy, including the globe. 

The differential diagnosis of such a peculiar condition should initially include cocaine abuse, fasciitis, or other opportunistic infections of an immunocompromised host, such as aspergillosis, herpes simplex, or herpes zoster. 

Dental practitioners should be aware of rhinocerebral mucormycosis, specifically in cases of diabetic and other immunocompromised patients. Atypical symptoms such as facial pain, sinus pain, or unexpected odontalgia of otherwise healthy teeth should alert clinicians. Moreover, when a patient seems to deteriorate after dental therapeutic interventions, one should consider rare conditions, such as mucormycosis, and promptly urge the patient to seek medical advice. Particularly, meticulous intraoral examination should be performed to all patients, as atypical lesions may thus be revealed and estimated. In the case presented, a slight change of the mucosal colour or dehiscence of the palate [15] might have been noticed. Similarly, it is imperative that all general dental practitioners note the patients' medical history and modify their diagnostic or therapeutic actions accordingly. As known, a raised glycated haemoglobin level is indicative of a poor blood glucose control by the diabetic patient [16]. If the dentist had required a serum glucose or glycated haemoglobin test, he might have estimated the poorly controlled diabetic condition and might have avoided the unnecessary extraction of the upper right third moral tooth. Instead, a more precise dental examination might have shown the need for further investigation of the patient's facial pain.

## Figures and Tables

**Table 1:** 

Laboratory findings	Initial values	Final values
Blood glucose (mg/dL)	197	110
Glycated haemoglobin (%)	12.3	5
CRP (mg/dL)	20.01	1
WBC (×10^3^)	9.47	5.14
Hemoglobin (g/dL)	10.3	12.4
Hematocrit (%)	25.8	37
